# The impact of early high-frequency ventilation uses in Brazilian preterm infants: an initiative to improve healthcare

**DOI:** 10.1590/1984-0462/2025/43/2024134

**Published:** 2025-10-20

**Authors:** Arthur de Andrade Oliveira, Taina Cristina Ferrari, Maurício Lauriano, Fábia Pereira Martins Celini, Anelise Roosch, Davi Casale Aragon, Walusa Assad Goncalves-Ferri

**Affiliations:** a Universidade de São Paulo, Faculdade de Medicina de Ribeirão Preto, Ribeirão Preto, SP, Brazil.

**Keywords:** High-frequency ventilation, Mechanical ventilation, Preterm infant, Developing countries, Ventilação de alta freqüência, Ventilação mecânica, Recém-nascido prematuro, Países em desenvolvimento

## Abstract

**Objective:**

High-frequency ventilation (HFV) is often used when conventional methods fail. Some studies suggest early HFV intervention might benefit infants with severe lung issues. This study compares early HFV at initial signs of respiratory distress to its later use when conventional ventilation fails.

**Methods:**

We conducted a retrospective cohort study on infants born weighing less than 1500 grams and with a gestational age under 28 weeks from January 2017 to December 2020. A guideline for early HFV was introduced in 2019. We analyzed two periods: late HFV (2017–2018), where HFV was applied after conventional ventilation failure (respiratory rate >60 rpm and driving pressure >20 cmH_2_O) to maintain pH >7.2 and PCO_2_ <60 mmHg; and early HFV (2019-2020), initiated when mean airway pressure exceeded 10 cmH_2_O and driving pressure >14 cmH_2_O.

**Results:**

Of the 139 infants studied, 98 received early HFV, while 41 had late. Early and late HFV groups had similar gestational ages (26.1±2.2 vs. 26.4±2.4 weeks, p=0.47) and birth weights (777±255 vs. 797±260 grams, p=0.66). Early HFV reduced mechanical ventilation duration with a hazard ratio of 0.66 (0.45–0.97) and was not linked to increased risks of hypoxemia, hypercapnia, or neurological issues. Mortality rates increased with late HFV, AdjRR [1.64 (1.05; 2.60)].

**Conclusions:**

Early HFV is effective for preterm infants with respiratory issues, reducing ventilation time and mortality. While results are promising, further randomized studies are essential to validate these findings and guide clinical practice.

## RESUMO

**Objetivo:**

A ventilação de alta freqüência (VAF) é frequentemente utilizada quando os métodos convencionais falham. Alguns estudos sugerem que a intervenção precoce com VAF pode beneficiar neonatos com comprometimento pulmonar grave. Este estudo compara a VAF precoce, iniciada nos primeiros sinais de desconforto respiratório, com sua aplicação tardia, após falha da ventilação convencional.

**Métodos:**

Estudo de coorte retrospectivo com neonatos nascidos com peso inferior a 1500 gramas e idade gestacional inferior a 28 semanas, entre janeiro de 2017 e dezembro de 2020. Analisamos dois períodos: VAF tardia (2017–2018), em que a VAF foi aplicada após falha da ventilação convencional (freqüência respiratória > 60 rpm e pressão de distensão > 20 cmH_2_O) para manter pH >7,2 e PCO_2_ <60 mmHg; e VAF precoce (2019-2020), iniciada quando a pressão média das vias aéreas excedeu 10 cmH_2_O e a pressão de distensão >14 cmH_2_O.

**Resultados:**

Dos 139 recém-nascidos estudados, 98 receberam VAF precoce e 41 VAF tardia. As idades gestacionais (26,1±2,2 vs. 26,4±2,4 semanas, p=0,47) e peso ao nascer (773±255 vs. 797±261 gramas, p=0,66) foram semelhantes entre os grupos. A VAF precoce reduziu a duração da ventilação mecânica (HR=0,66 [0,45–0,97] sem aumento de hipoxemia, hipercapnia ou complicações neurológicas. A taxa de morte RRAj [1,64 (1,05; 2,60)].

**Conclusões:**

A VAF precoce mostra-se eficaz na redução do tempo de ventilação e da mortalidade em prematuros, embora sejam necessários estudos randomizados para confirmação.

## INTRODUCTION

 High-frequency ventilation (HFV) uses small tidal volumes, and many articles show the benefits associated with this ventilatory mode. However, there are doubts about the best time to install the HFV in infants.^1-4^

 Early use of HFV in preterm infants is poorly documented in low/middle-income countries, settings with specific characteristics such as inadequate nutrition, high infection rate, and low use of antenatal corticosteroids. The data from high-income countries show that HFV is feasible and that early use can present benefits.^5,6^

 The experience with HFV is used in specific situations, such as rescue ventilation mode due to conventional ventilation (CV) failure;^7^ on the opposite side, some articles proposed HFV as the first ventilation mode, showing favorable outcomes.^8^

 When the CV fails, high driving pressure is needed, so the patient presents unfavorable clinical conditions due to hours of aggressive ventilation, probably associated with acidosis, poor cardiac conditions, and metabolic instability, increasing the possibility of HFV failure.^3,7^

 HFV was initially employed as a rescue strategy in cases of failure of conventional mechanic ventilation;^7^ however, since 2019, we have followed the early HFV proposal as a protective ventilation mode, implementing the use in the first signs of ventilatory worsening^9,10^ aimed to improve outcomes related to mechanical ventilation. Our study compares the outcomes of early versus late HFV use in preterm infants. 

## METHOD

 We designed a retrospective cohort study from January 2017 to December 2020 to evaluate the outcomes related to HFV use in preterm infants. The data were collected through a retrospective collection. Two investigators reviewed the data to confirm the threshold when the HFV was implemented — the study following the Strengthening the Reporting of Observational Studies in Epidemiology (STROBE) checklist. 

 All patients weighing less than 1500 grams and <28 weeks, with respiratory distress syndrome (RDS),^11^ born 2017–2020, that used HFV during their hospitalization were included. Thus, the patients were divided into two groups according to the indication criteria for HFV:

- Late HFV group (rescue HFV use/2017–2018) was indicated by CV failure (RR >60 rpm and driving pressure >20 cmH_2_O).^9^
- Early HFV group (protective HFV use/2019–2020), those who received the early indication of HFV according to the institutional guideline: HFV used when the mean airway pressure (MAP) >10 cmH_2_O and driving pressure >14 cmH_2_O.^12^
- Ventilatory parameters were established to maintain PH >7.2 and PCO_2_ < 60 mmHg.

Exclusion criteria: major malformations and genetic syndromes. 

 The local guidelines for HFV use were modified in 2019 after a staff consensus about HFV use; we followed the articles that recommended early use of HFV according to the first signs of ventilatory worsening^12-14^ and also drew on the most recent articles about mortality by lung injury.^15^ So, the local guidelines since 2019 recommended HFV use when MAP ≥10 and driving pressure ≥14 cmH_2_O.^14^ Before 2019 the local guidelines recommended HFV use after CV failure.^8-10^

 CV was the initial choice for all patients, provided by mechanical ventilators cycled on time and pressure controlled. Target tidal volumes were 4–6 mL/kg, and positive end-expiratory pressure (PEEP) was 6-9 mmHg. The mechanical ventilators were used in servo I (Maquet, Getinge AB, Sweden). To perform the HFV, we used HFFI/Babylog 8000 (Drager, German). Patients under 72 hours who showed a fraction of oxygen, more than 30%, received surfactant 200 mg/kg. 

 The assistant physician adjusted the ventilator parameters to maintain arterial oxygen saturation, measured by pulse oximetry, between 91 and 95%, PaO_2_ between 50 and 80 mmHg, PaCO2between 40 and 60 mmHg, and pH between 7.20 and 7.40. Arterial blood gas analysis was collected immediately before the indication of HFV and after one hour of its installation. 

 The initial parameters of HFV were map 10–12 cm H_2_O, frequency 10 HZ, and amplitude 100%. Ventilatory management was established according to international recommendations.^12,14,15^

 The variables evaluated to characterize the cohort were: gestational age (weeks) and birth weight (grams) in the HFV implementation, antenatal steroid exposition, early sepsis (clinical), hypothermia at NICU admission (<36.5°C), hemodynamic status (blood pressure — systolic/diastolic), lactate (1 mg/dl), excess base, blood gas (pH, PCO_2_, PaO_2_), ventilatory parameters (MAP, driving pressure), SNAPPE II, PaO_2_/FiO_2_ and OI. 

 The outcomes were: pneumothorax, pulmonary hemorrhage, bronchopulmonary dysplasia (36 weeks postmenstrual age), extubation failures, mechanical ventilation days (considering conventional and HFV days), death, peri-intraventricular hemorrhage (PIVH III or IV) according to Volpe classification.^16^

 To estimate relative risks, simple and multiple log-binomial regression models were adjusted, considering sex, gestational age, and use of antenatal corticosteroids as covariates. To compare the groups about CV and HFV times, hazard ratios were estimated using the multiple Cox proportional hazards model, considering the same covariates mentioned above. We used Wilcoxon’s non-parametric test to compare perinatal variables between groups. To estimate the differences between group means concerning the changes in the variables of interest, Bayesian multiple linear regression models were adjusted, taking into account the same covariates previously mentioned. The analyses were conducted using R version 4.0.5 and SAS version 9.4. The significance level was set at 5% 

 This study was approved by the Institutional Review Board of Ribeirão Preto Medical School, University of São Paulo (FMRP-USP, Certificate of Presentation for Ethical Appreciation — CAAE number 33541620.8.000.5440, approval number 4.164.064). The study followed the STROBE guidelines. The parents were informed about the treatment on institutional guidelines. 

## RESULTS

 In the study period, 510 preterm infants weighing less than 1500 g and having less than 28 gestational weeks were born at the hospital; 356 infants did not use HFV. One hundred thirty-nine (139) children were included (used HFV), 98 were submitted to early HFV, and 41 used late HFV. Fifteen infants were excluded due to malformations. 

 Early and late HFV groups did not present differences in birth weight, gestational age, and SNAPPEII values (Table 1). The steroids antenatal use was, in the early and late HFV group, respectively, 73.1 vs. 75.5% (p=0.83), the early sepsis rate 48.4 vs. 41.3% (p=0.65), and hypothermia at NICU admission was 60.8 vs. 58.5% (p=0.85). 

**Table 1:** Perinatal variables according to group.

Variables	Early HFV n=98	Late HFV n=41	p-value
Mean gestational age (birth/weeks)	26.1±2.2	26.4±2.4	0.471
Birth weight (g)	777.3±254.6	797,4±260.3	0.664
SNAPPE 2	41.17±21.8	56.1±31,9	0.082
Mean gestational age (HFV use/weeks)	26.7±2.8	27.0±3.1	0.598
Antenatal steroids (%)	73.1	75.5	0.831
Hypothermia at NICU admission (%)	60.8	58.5	0.853

HFV: high-frequency ventilation.

 We observed that the initial respiratory parameters were similar between the early and late groups, respectively: MAP 11.2 cmH_2_O (standard deviation — SD 1.8) vs. 12.9 cmH_2_O (SD 2.2); frequency 8.7 Hz (SD 1.7) vs. 8.2 Hz (SD 1.2); amplitude 92% (SD 0.19) vs. 94% (SD 0.18), gestational age in the first day of HFV use 26,7 (SD 2,8) vs. 27,0 weeks (SD 3,1) (Table 2). 

**Table 2:** Comparison between cardio-respiratory values within groups before and after one hour of high-frequency ventilation implementation.

	Early HFV (n=98)		Late HFV (n=41)		Late - Early HFV (mean difference)	LI 95%CI	UI 95%CI
	Conventional ventilation	HFV early use	Conventional ventilation	HFV late use			
pH	7.18±0.14	7.26±0.17	7.18±0.14	7.25±0.16	-0.002	-0.081	0.078
PO_2_	68.6±21.7	77.4±46.6	82.3±42.3	75.1±49.1	-16.74	-35,99	1.76
PCO_2_	55.6±23.0	40.3±18.3	53.9±21.1	43.2±17.9	1.844	-10.37	14.422
Bicarbonate	19.9±4.73	16.9±5.2	19.0±4.2	17.4±5.4	1.43	-0.357	3.153
Lactate	4.4±3.2	6.0±4.2	5.7±4.0	7.7±6.0	-0.11	-1.84	1.66
PaO_2_/FiO_2_	147.7±88.3	185.8±144.5	143 ±101.3	178.4±154.3	0.31	-52.67	55.61
OI	10.0±6.3	11.1±9.1	12.8±9.4	15.6±15.1	-0.45	-5.91	4.97
Systolic BP	49.7±12.16	49.1±9.99	51.6±11.5	48.0±11.0	0.32	-7.7	8.31
Diastolic BP	32.1±11.3	31.9±9.3	34.1±10.9	31.1±10.4	0.14	-7.78	8.05
Medium BP	28.0±11.7	28.6±9.9	27.2±10.0	26.1±11.7	-1.2	-8.89	6.81

HFV: high frequency ventilation; LI: lower Interval; UI: upper interval; 95%CI: 95% confidence interval; OI: oxygenation index; BP: blood pressure. Adjusted for gender, gestational age, and use of antenatal steroids (Bayesian multiple linear regression models).

 Concerning metabolic and hemodynamic changes, we observed similarities in the blood gas variables in both groups; also, blood pressure values remained stable (Table 2). 

 When the laboratory values were analyzed, both groups presented a reduction of PCO_2_ and stability of PO_2_. Regarding the pH value, we noticed that both approaches showed subtle changes in the initial and final values. 

 In Table 3, we observed that HFV was not related to worse pulmonary or neurological outcomes. 

**Table 3:** Simple and multiple log-binomial regression models comparing the neurological and pulmonary outcomes between the early and late high frequency ventilation groups.

	Early HFV n=98 (%)	Late HFV n=41 (%)	AdjRR (95%CI)
Pneumothorax	18 (18.3)	9 (21.9)	1.23 (0.55–2.74)
Pulmonary hemorrhage	32 (32.6)	11 (28.8)	0.82 (0.41–1.63)
BPD (28 days)	15 (35.7)	5 (38.4)	1.19 (0.42–3.30)
BPD moderate/severe	8(22.8)	1(11.1)	0.92 (0,47–1,80)
Extubation fail	25 (36.7)	6 (28.5)	0.78 (0,32–1,91)
Death	25 (25.5)	18 (45)	1.64 (1.05–2.60)
PIVH	9 (40.9)	1 (25)	0.89 (0.34–2.34)

HFV: high frequency ventilation; AdjRR: adjusted relative risk; 95%CI: 95% confidence interval; BPD: bronchopulmonary dysplasia. PIVH: peri-intraventricular hemorrhage. Adjusted for gender, gestational age, and use of antenatal steroids.

 Regarding mortality among the groups, early HFV was not associated with an increase in the death rate. 

**Figure 1:**
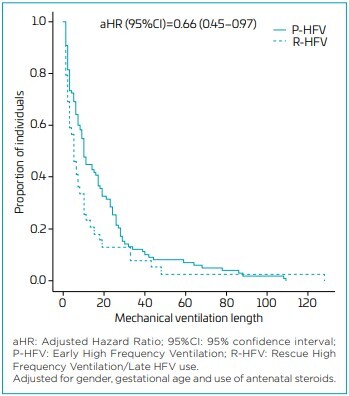
Mechanical ventilation length of stay according to group.

 Regarding the mechanical ventilation days, we can observe that the patients submitted to early HFV presented shorter mechanical ventilation days, with adjusted hazard ratio=0.66 (0.45–0.97) (Figure 1). 

## DISCUSSION

 Our study found benefits associated with the early indication of HFV based on driving pressure values, such as a reduction of mechanical ventilation length of stay. HFV has been used in neonatology for decades; however, there are some questions about the best time to install HFV in premature patients and on the feasibility of the use in resource-limited settings.^17-22^ Concerns about early use include an excessive variation of PCO_2_, unfavorable neurological outcomes, and the need for sedation; also, the restricted indication of HFV has impaired its use in LMICs; thus, HFV is used frequently as rescue ventilation; however, the concern is the lower cardiac capacity of sick preterm infants, possible hemodynamic instability, and low tolerance to HFV.^21-24^

 Regarding HFV viability, Prashant et al.^5^ conducted a study on HFV in India. However, infants weighing less than 750 g were excluded. The authors demonstrated that elective HFV in preterm infants is feasible, even in developing countries, associated with early oxygen weaning, consequently reducing treatment costs.^5,6,25^ Our study evaluated infants with gestational age <27 weeks, weight <800 g, and high mortality score (SNAPEII mean=40), who showed a massive rate of hypothermia at NICU admission (60%), and an antenatal steroids rate of around 70%. In the literature, there are few articles about the early use of HFV in infants with these characteristics, mainly in LMICs.^5,25^ Our data is crucial to resource-limited settings since the sample studied (small and sick preterm infants) is frequent in developing countries 

 Studies were performed comparing early and elective HFV with late HFV, demonstrating a slight effect on early use when considering bronchopulmonary dysplasia (BPD) and mortality rates. However, meta-analysis results are inconclusive because ventilatory management in HFV was very different.^22^ Salvo et al. exposed a different proposal, implemented HFV use before 2 hours of life in the preterm infants that did not receive antenatal steroids, demonstrating an association with favorable outcomes.^25^ This is promising since there are low rates of antenatal steroids, mostly in resource-limited settings.^26^ After 2000, eight articles considered lung disease severity to indicate HFV, and six showed HFV benefits.^10,27^ Three articles showed shorter mechanical ventilation length.^14,15,25^ These data are comparable with our results. We demonstrated the absence of adverse effects related to the early use of HFV since there was no occurrence of hypoxemia, hypercapnia or hypocapnia, hemodynamic instability, or unfavorable neurological outcomes. 

 Our guideline was based on a proposal for HFV use based on avoiding aggressive ventilation, using the high levels of driving pressure (>14 cmH_2_O) in CV as the threshold. Driving pressure has been indicated as an important parameter associated with lung injury and unfavorable outcomes.^13^ On HFV impact after 1 hour, we can observe that both strategies produced similar improvement of the PCO_2_, PO_2_, PO_2_/FiO_2_ ratio, and OI. We also did not observe a difference in PIVH, pneumothorax, death or pulmonary hemorrhage, reinforcing the literature data indicating that HFV is a safe therapy, even with the early use.^27^

 We did not observe any difference in the bronchodysplasia rate. Perhaps this fact may be attributed to the higher mortality rate in the late HFV group before 36 weeks. Our study, while valuable, does have some limitations. The cardiac evaluation was primarily based on blood pressure and biomarkers, which ideally should be supplemented with echocardiographic assessment. Additionally, we utilized HFFI/Babylog 8000, a device associated with a higher risk of unfavorable outcomes compared to oscillatory mode ventilation. Nonetheless, it is important to recognize that modern devices, while often more effective, tend to be prohibitively expensive, leading many resource-limited countries to continue using HFFI. 

 Another consideration is the change in indications for HFV and the comparison of two different time periods. However, the main characteristics of the preterm infants were similar, demonstrating that the groups are comparable. These limitations emphasize the need for further research in this area, which could lead to future advancements. 

 There have yet to be any publications about HFV interrupter flow (HFFI).^2,4,28^ Nevertheless, many hospitals continue using HFFI to perform HFV due to the high cost of modern ventilators.^5,6^ Due to the lack of data about HFFI, there are many doubts regarding its use, and our data provide essential information to neonatologists working in resource-limited settings. Therefore, we believe that the limitations do not impair the importance of our results on the early use of HFV in preterm infants. Even though most countries do not have oscillatory ventilators, the use of HFFI is also associated with benefits for preterm infants. We used the STROBE checklist, which improves the quality of the report and facilitates the replication of the study, clarifying aspects such as views, conflict of interest, and generalizability of results. Therefore, we recommend using early HFV as a protective ventilatory strategy for preterm infants since data has demonstrated that HFV decreases pulmonary injury to severe lung disease.^2^ We showed the feasibility and essential benefits of HFV use for Brazilian preterm infants. Our results are important for preterm infants born worldwide; however, this is mainly true in developing countries where small and sick infants are usual. 

 In conclusion, early HFV shows potential for managing respiratory issues in preterm infants, which could lead to improved care. Our study compared early HFV with late HFV and found an association between early HFV use and a shorter duration of mechanical ventilation, indicating possible benefits. Also, the data shows a decrease in the number of deaths. Early HFV may be a promising option. However, it is important to understand that this field is still evolving. Further research must confirm these benefits and establish a solid evidence base for definitive clinical recommendations. 

## Data Availability

The database that originated the article is available with the corresponding author
